# Reelin Increases the Sphingomyelin Content of the Plasma Membrane and Affects the Surface Expression of GPI‐Anchored Proteins in Hippocampal Neurons

**DOI:** 10.1111/jnc.70225

**Published:** 2025-09-03

**Authors:** Yuto Takekoshi, Hugo Ando, Takao Kohno, Hiroshi Takase, Tomohiko Taguchi, Makoto Arita, Toshihide Kobayashi, Mitsuharu Hattori

**Affiliations:** ^1^ Department of Biomedical Science Graduate School of Pharmaceutical Sciences, Nagoya City University Nagoya Aichi Japan; ^2^ Core Laboratory Graduate School of Medical Sciences, Nagoya City University Nagoya Aichi Japan; ^3^ Laboratory of Organelle Pathophysiology, Department of Integrative Life Sciences Graduate School of Life Sciences, Tohoku University Sendai Miyagi Japan; ^4^ Laboratory for Metabolomics RIKEN Center for Integrative Medical Sciences Yokohama Japan; ^5^ Molecular and Cellular Epigenetics Laboratory Graduate School of Medical Science, Yokohama City University Yokohama Japan; ^6^ Division of Physiological Chemistry and Metabolism Graduate School of Pharmaceutical Sciences, Keio University Tokyo Japan; ^7^ Human Biology‐Microbiome‐Quantum Research Center (WPI‐Bio2Q) Keio University Tokyo Japan; ^8^ Laboratoire de Bioimagerie et Pathologies UMR 7021 CNRS, Faculté de Pharmacie, Université de Strasbourg Illkirch France

**Keywords:** membrane, neuron, reelin, sphingomyelin

## Abstract

Sphingomyelin (SM) is primarily located in the outer leaflet of the plasma membrane. It plays a crucial role in intercellular communication and the morphology of neuronal cells by influencing the localization and function of various proteins. However, the mechanisms regulating the SM content in the neuronal plasma membrane remain largely elusive. In this study, we discovered that Reelin, an important secreted signaling protein in the central nervous system, increases the SM content of the plasma membrane of cultured hippocampal neurons by promoting SM synthesis using a SM specific probe and a fluorescently labeled SM precursor molecule. This increase in SM was associated with increased surface expression of glycosylphosphatidylinositol (GPI)‐anchored proteins analyzed by immunohistochemistry or using phosphatidylinositol‐specific phospholipase C, suggesting a functional link between SM levels and membrane protein trafficking. Furthermore, comparative lipidomic analysis of the postsynaptic density fraction by LC–MS/MS revealed distinct alterations in SM‐related lipid species between wild‐type and Reelin‐deficient mice. These findings suggest that Reelin regulates the SM content in the neuronal plasma membrane, which, in turn, affects the function and morphology of the neuron by affecting the surface levels of GPI‐anchored proteins. These findings identify a novel role for Reelin in modulating neuronal membrane lipid composition, which may underlie its diverse functions in neuronal development and synaptic plasticity in both the developing and adult brain.

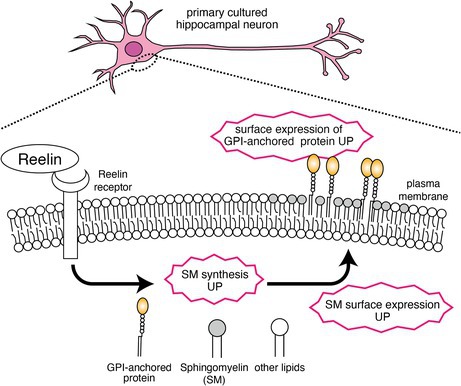

## Graphical abstract

We identified a previously unrecognized mechanism by which the extracellular protein Reelin increases sphingomyelin synthesis in hippocampal neurons, increasing plasma membrane sphingomyelin, and GPI‐anchored proteins. This coupling of extracellular signaling to membrane lipid remodeling provides new insight into how Reelin shapes neuronal surface composition, with potential implications for synaptic function and neurodevelopmental disorders.

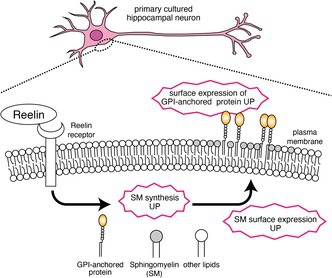

**Abbreviations**
ApoER2: apolipoprotein E receptor 2
BSA: bovine serum albumin
Cer: ceramide
CNTN‐1: contactin‐1
CTb: cholera toxin subunit B
DAG: diacylglycerol
EDTA: ethylenediaminetetraacetic acid
Eqt II: Equinatoxin II
FBS: fetal bovine serum
GPI: glycosylphosphatidylinositol
IQR: interquartile range
LC: liquid chromatography
Lys: lysenin
MS: mass spectrometry
PBS: phosphate‐buffered saline
PC: phosphatidylcholine
PE: phosphatidylethanolamine
PFA: paraformaldehyde
PI: phosphatidylinositol
PLC: phospholipase C
PLL: poly‐L‐lysine
PS: phosphatidylserine
PSD: postsynaptic density
RRID: research resource identifier
SDS: sodium dodecyl sulfate
SM: sphingomyelin
SMS: sphingomyelin synthase
TBST: tris‐buffered saline with 0.05% Tween 20
TG: triglyceride
TLC: thin‐layer chromatography
UPLC: ultraperformance LC
VLDLR: very low‐density lipoprotein receptor
WT: wild‐type

## 1 Introduction

The plasma membrane is composed of a diverse array of lipid species that not only define cellular boundaries but also organize membrane microdomains and regulate the localization and function of membrane‐associated proteins. Among these lipids, sphingomyelin (SM) is one of the most abundant sphingolipids in mammalian cells and is predominantly localized to the outer leaflet of the plasma membrane (Zachowski 1993; Kobayashi and Menon 2018). SM contributes to the formation of lipid rafts—cholesterol‐ and sphingolipid‐enriched microdomains—that facilitate the clustering of signaling molecules and glycosylphosphatidylinositol (GPI)‐anchored proteins (Lingwood and Simons 2010; Paulick and Bertozzi 2008). In neurons, SM is involved in a variety of essential functions, including compartmentalization of receptor proteins, modulation of signal transduction pathways (Hering et al. 2003; Wheeler et al. 2009), and dendritic spine development (Arroyo et al. 2014). Moreover, alterations in the SM‐to‐ceramide (Cer) ratio have been implicated in the pathogenesis of neurodegenerative disorders such as Alzheimer's disease (Yin 2023), underscoring the importance of SM in maintaining neuronal homeostasis. However, the mechanisms that regulate SM levels in neuronal membranes remain poorly understood.

Reelin is a large, secreted extracellular glycoprotein predominantly expressed in the central nervous system, where it plays essential roles in neuronal migration, dendritic development, synaptic plasticity, and neurodegeneration (Cuchillo‐Ibañez et al. 2016; Jossin 2020; Alexander et al. 2023; Reive et al. 2024; Hattori 2025). Reelin exerts its effects by binding to apolipoprotein E receptor 2 (ApoER2) and very low‐density lipoprotein receptor (VLDLR), both of which are members of the low‐density lipoprotein receptor family (Trommsdorff et al. 1999; Jossin 2020; Alexander et al. 2023; Hattori 2025). This ligand‐receptor interaction initiates a signaling cascade involving the phosphorylation of the intracellular adaptor protein Dab1 by Src family kinases, including Fyn (Bock and Herz 2003). These pathways are crucial not only during brain development (Sekine et al. 2014) but also in maintaining synaptic function in the adult brain (Herz and Chen 2006; Stranahan et al. 2013; Alexander et al. 2023). Notably, reduced Reelin signaling has been observed in patients with neurodevelopmental and neurodegenerative disorders, such as schizophrenia and Alzheimer's disease (Folsom and Fatemi 2013; Katsuyama and Hattori 2024; Krstic et al. 2013; Cuchillo‐Ibañez et al. 2016; Faini et al. 2021; Reive et al. 2024). Therefore, it is urgent to reveal how Reelin demonstrates versatile biological functions to establish a novel treatment for these diseases.

Our previous lipidomic analysis revealed that the cerebral cortex of Reelin‐deficient (*reeler*) mice displays an altered lipid profile compared to wild‐type (WT) controls at embryonic day 17.5 (E17.5) (Mizukami et al. 2018). Given that the embryonic cortex at this stage consists predominantly of neurons with minimal glial contribution, these findings strongly suggest a neuron‐specific role for Reelin in lipid regulation. Furthermore, some studies have demonstrated that Fyn activation can increase SM content in the plasma membrane of non‐neuronal cells (Baba et al. 2009; Kim et al. 2018), raising the possibility that Reelin signaling may regulate neuronal SM levels via a similar mechanism. Based on these observations, we hypothesized that Reelin influences SM metabolism in neurons, thereby modulating membrane composition and associated protein localization.

In this study, we demonstrate that Reelin signaling enhances SM content in the plasma membrane of cultured hippocampal neurons by promoting SM biosynthesis. This effect is accompanied by increased surface expression of GPI‐anchored proteins, including Thy‐1 and contactin‐1. Moreover, comparative lipidomic analysis of the postsynaptic density (PSD) fraction in WT and *reeler* mice revealed alterations in lipid species consistent with impaired SM synthesis. Together, these findings uncover a novel function of Reelin in regulating neuronal membrane lipid composition, providing new insights into the molecular basis of its multifaceted roles in brain development and function.

## 2 Materials and Methods

### 2.1 Animal Care

All animal procedures were approved by the Animal Care and Use Committee of Nagoya City University and conducted in accordance with the Institutional Guidelines for Animal Experimentation (Approval Number: 23‐001H05). Mice were housed under a 12‐h light/dark cycle (lights on from 6:00 A.M.–6:00 P.M.) and provided with standard chow and water ad libitum. Mice were housed in a temperature‐controlled room at 23.5°C ± 2.5°C and 52.5% ± 12.5% relative humidity. Mice were housed in groups of no more than 5 per cage. Crl:CD‐1 mice (RRID: MGI:5652673) were obtained from Japan SLC. *Reeler* mice (B6C3Fe a/a‐Reln^rl^/J, RRID: IMSR_JAX:000235) were obtained from The Jackson Laboratory and backcrossed to the Crl:CD‐1 strain. Both male and female mice were used in all experiments, and no sex‐dependent differences were observed in the results reported in this study. Adult WT and *reeler* mice ranging from 30 to 37 g and 12 to 30 g in body weight, respectively, were used. Pregnant dams and adult mice were deeply anesthetized with isoflurane gas (Wako, cat. no. 095–06573) and euthanized by cervical dislocation. Embryos at E17.5 were collected immediately following maternal euthanasia and were euthanized by decapitation. Neonatal mice at P0 were euthanized by rapid decapitation.

### 2.2 Cell Culture and Transfection

HEK293T (RRID:CVCL_0063) cells were cultured as previously described (Kohno et al. 2015; Nakano et al. 2007). According to the International Cell Line Authentication Committee database, HEK293T cells are not listed among commonly misidentified cell lines. For preparation of mock‐ or Reelin‐containing medium, HEK293T cells were transfected using Polyethylenimine “Max” (Polysciences, cat. no. 24765) according to the manufacturer's instructions. The pcDNA3.1/myc‐HisA vector (Thermo Fisher Scientific, cat. no. V80020) was used for mock transfection. 5 h post‐transfection, the medium was replaced with Neurobasal medium (Thermo Fisher Scientific, cat. no. 21103049) containing penicillin–streptomycin (Nacalai Tesque, cat. no. 09367‐34). After 3 days, the culture supernatant was collected, filtered, and stored at 4°C until use. The concentration of Reelin in the supernatant was approximately 3 nM, determined by western blotting through comparison with purified Reelin of known concentration (Ishii et al. 2023a). Before addition to cultured neurons, the supernatant was supplemented with 2% B27 (Thermo Fisher Scientific, cat. no. 17504044) and 2 mM GlutaMAX (Thermo Fisher Scientific, cat. no. 35050061). In preparing mock‐ or Reelin‐containing media, the passage number of HEK293T cells was not strictly controlled; instead, cell identity was verified by monitoring characteristic morphology and growth rate, and the Reelin concentration was measured for each preparation.

### 2.3 Primary Culture of Mouse Neurons

The primary‐cultured hippocampal neurons and cerebral cortical neurons were prepared as previously described (Ishii et al. 2023b; Kohno et al. 2009; Ogino et al. 2020). Primary neurons were obtained from embryonic and neonatal mice. For embryonic cultures, 17 pregnant mice were used, each providing 8–16 embryos. For neonatal cultures, 5–12 postnatal day 0 (P0) pups were used per preparation, with 12 independent preparations in total. For each sample, brains from embryos/pups were pooled before plating. Hippocampi and cerebral cortex were dissected from E17.5 to P0 mice and digested with 0.25% trypsin (Thermo Fisher Scientific, cat. no. 25200056) at 37°C for 5 min. Tissue was gently triturated using a fire‐polished Pasteur pipette and washed twice with Neurobasal medium. The dissociated neurons were counted and seeded onto poly‐L‐lysine (PLL, 0.01%, Sigma‐Aldrich, cat. no. P1399)‐coated glass coverslips or plates in Neurobasal medium supplemented with 2% B27, 2 mM GlutaMAX, and penicillin–streptomycin. For neurons from *reeler* mice, genotypes were rapidly determined by PCR (Koie et al. 2014) prior to dissection. To evaluate Reelin‐specific effects on SM content, GPI‐anchored proteins, and phosphorylation, *reeler*‐derived neurons were used to avoid interference by endogenous Reelin. Seeding densities were as follows: 1.25–2.5 × 10^3^ cells/cm^2^ for SM staining and immunocytochemistry; 5 × 10^4^ cells/cm^2^ for SM synthesis assays; 8.75–12.5 × 10^3^ cells/cm^2^ for phosphatidylinositol‐specific phospholipase C treatment.

### 2.4 Antibodies and Reagents

The following antibodies were purchased commercially: mouse monoclonal anti‐Reelin G10 (Merck Millipore, cat. no. MAB5364, RRID:AB_2179313), mouse monoclonal anti‐PSD95 (Santa Cruz Biotechnology, cat. no. sc‐32 290, RRID:AB_628114), rabbit monoclonal anti‐Synaptophysin (Cell Signaling Biotechnology, cat. no. 5461, RRID:AB_10698743), mouse monoclonal anti‐β‐actin (Sigma‐Aldrich, cat. no. A1978, RRID:AB_476692), rabbit polyclonal anti‐Thy1 (Bioss, cat. no. bs‐0778R, RRID:AB_10857837), mouse monoclonal anti‐Tuj1 (R&D Systems, cat. no. MAB1195, RRID:AB_357520), rabbit monoclonal anti‐phospho‐tyrosine mix (cell signaling Biotechnology, cat. no. 8954, RRID:AB_2687925), mouse monoclonal anti‐Myc (9E10, Sigma‐Aldrich, RRID:AB_260581), goat polyclonal anti‐Contactin1 (R&D Systems, cat. no. AF904, RRID:AB_2292070). Alexa 594 conjugated cholera toxin subunit B (cat. no. C34777) and BODIPY TR ceramide complexed to BSA (cat. no. B34400) were purchased from Thermo Fisher Scientific. ECL Anti‐mouse IgG, horseradish peroxidase linked F(ab')_2_ fragment (from sheep) (Cytiva, cat. no. NA9310, RRID:AB_772193), ECL Anti‐rabbit IgG, horseradish peroxidase linked F(ab')_2_ fragment (from sheep) (Cytiva, cat. no. NA9340, RRID:AB_772191), and peroxidase AffiniPure donkey anti‐goat IgG (H + L) (Jackson ImmunoResearch Laboratories, cat. no. 705–035‐003, RRID:AB_2340390) were used for secondary antibodies for western blotting.

### 2.5 Plasmids

The expression plasmid for SM synthase 2 (SMS2) in pcDNA3.1(+)/Myc‐His was previously described (Kidani et al. 2012). The coding region, including the Myc epitope and His tag, was subcloned into the pCAGGS vector (Niwa et al. 1991). The Reelin expression plasmid (pCrl) and pCAGGS‐EGFP were kindly provided by Prof. T. Curran (D'Arcangelo et al. 1997) and by Prof. K. Nakajima (Honda and Nakajima 2006), respectively. Expression plasmids for Equinatoxin II‐GFP (EqtII‐GFP, #RDB13496) and Venus‐Lysenin (Venus‐Lys, #RDB13959) were described previously (Kiyokawa et al. 2004, 2005; Yachi et al. 2012; Yamaji‐Hasegawa et al. 2003; Makino et al. 2015). Previously described custom‐made materials are available from the original authors as indicated in the cited publications. The newly generated plasmid construct will be shared upon reasonable request.

### 2.6 Purification of Recombinant Protein

EqtII‐GFP and Venus‐Lys were purified as previously described (Bhat et al. 2013) with minor modifications. Briefly, recombinant proteins were expressed in *E. coli* BL21 CodonPlus (DE3)‐RIL cells (Agilent Technologies, cat. no. 230245) by induction with 0.4 mM isopropyl β‐D‐1‐thiogalactopyranoside at 25°C overnight. Cells were harvested by centrifugation at 4000 × *g* for 2 min at 4°C. Pellets were resuspended in binding buffer (20 mM sodium phosphate, pH 7.4; 500 mM NaCl; 20 mM imidazole) and lysed by sonication. Insoluble debris was removed by centrifugation at 7300 × *g* for 10 min at 4°C. Supernatants were applied to Ni Sepharose 6 Fast Flow resin (Cytiva, cat. no. GE17‐5318‐01) and purified according to the manufacturer's instructions. Proteins were eluted using elution buffer (20 mM sodium phosphate, pH 7.4; 500 mM NaCl; 500 mM imidazole).

### 2.7 Western Blotting

Western blotting was performed as previously described (Nakano et al. 2007). In brief, samples were separated by SDS‐PAGE (8.5% acrylamide gel for SMS2 phosphorylation assay and 10% for others) with Precision Plus Protein All Blue Prestained Protein Standards (Bio‐Rad, cat. no. 1610373) as molecular weight markers. In each experiment, all samples were treated identically, and we did not measure protein concentration before gel loading. We confirmed that equal protein amounts were applied between the samples being compared, and β‐actin was used as a loading control whenever possible. Proteins were then transferred to a polyvinylidene fluoride membrane (Merck, cat. no. IPVH00010) using Trans‐Blot SD Semi‐Dry Electrophoretic Transfer Cell (Bio‐Rad, cat. no. 170‐3940). The membrane was incubated with 2% BSA and 3% skimmed milk in Tris‐buffered saline with 0.05% Tween 20 (TBST) for SMS2 phosphorylation assay and others, respectively. The membrane was then incubated with the primary antibody for more than 2 h at room temperature, washed three times with TBST, incubated with the peroxidase‐conjugated secondary antibody for 1 h at room temperature, and washed four times with TBST. The bands were then visualized with Immobilon Chemiluminescent HRP Substrate (Sigma Aldrich, cat. no. WBKLS0500). Images were captured using LAS4000‐Mini (Fuji, Tokyo, Japan) and analyzed with Fiji/Image J software (National Institutes of Health, RRID:SCR_002285).

### 2.8 Staining of SM in the Plasma Membrane

Neurons were briefly rinsed with phosphate‐buffered saline (PBS) and fixed with 4% paraformaldehyde (PFA) in PBS at room temperature for 10 min. After washing with PBS, neurons were incubated with 3% BSA in PBS for 1 h, followed by incubation with either Venus‐Lys (1–2 μg/mL) or EqtII‐GFP (0.3 μg/mL) for 1 h at room temperature. After staining, cells were washed three times with PBS. For subsequent immunostaining, samples were post‐fixed with 4% PFA in PBS, permeabilized with 0.1% Triton X‐100 in PBS (PBST) for 1 h, and incubated with primary antibodies diluted in PBST containing 3% BSA at room temperature for several hours. After washing with PBST, samples were incubated with Alexa Fluor 594‐conjugated secondary antibodies (1:500, Life Technologies, cat. no. A21203) and Hoechst 33342 (2 μg/mL, Life Technologies, cat. no. H3570) for 1 h, followed by PBS washing. Fluorescence images were acquired using a BZ‐X710 microscope (Keyence, RRID:SCR_017202).

### 2.9 SM Synthesis Assay

Primary cultured neurons were treated with control or Reelin‐containing medium containing 25 μM BODIPY‐Ceramide for 1 h in a CO_2_ incubator. After incubation, lipids were extracted by the Bligh & Dyer method (Bligh and Dyer 1959) from the neurons and applied to thin‐layer chromatography (TLC) plates (Merck, cat. no. 113794) with commercially obtained SM (Cayman Chemical, cat. no. 22674) as a standard. The samples were developed with chloroform, methanol, and ammonia solution (65:35:8, *v*/*v*) as solvent. BODIPY and standard SM were visualized by Typhoon 9400 scanner (Cytiva) and 0.005% primulin (MP biomedicals) solution, respectively. The relative amount of BODIPY‐SM (normalized to total BODIPY‐labeled lipids) was quantified using Fiji/ImageJ and ImageQuant TL ver.7 software (Cytiva, RRID:SCR_018374).

### 2.10 Phosphatidylinositol‐Specific Phospholipase C (PI‐PLC) Treatment

Primary cultured hippocampal neurons were treated with control or Reelin‐containing medium for 1 h. Following this treatment, the culture medium was replaced with neurobasal medium containing 0.2 U/mL PI‐PLC (Sigma‐Aldrich, cat. no. P5542) for 1 h. Both the cell lysate and the culture supernatant were prepared in SDS sample buffer (the final concentration: 62.5 mM Tris–HCl pH 6.8, 10% glycerol, 2% SDS, 5% 2‐mercaptoethanol, 0.05% bromophenol blue) and analyzed by western blotting.

### 2.11 Nucleofection and Immunoprecipitation

Nucleofection was performed as previously described (Kohno et al. 2020). Cortical neurons from *reeler* mice were resuspended in 95 μL of supplemented Nucleofector solution (Lonza, cat. no. VPG‐1001) containing 5 μg of either pCAGGS‐SMS2‐Myc or pCAGGS‐EGFP. The cell suspension was transferred to a cuvette and electroporated using a Nucleofector II device (Lonza). Neurons were then transferred to Neurobasal medium supplemented with 10% FBS and plated on PLL‐coated dishes. 2–3 h later, the medium was replaced with Neurobasal medium containing 2% B27 and 2 mM GlutaMAX. For immunoprecipitation, neurons were lysed in buffer containing 25 mM Tris–HCl (pH 7.8), 10 mM sodium pyrophosphate, 2 mM sodium orthovanadate, 1 mM EGTA, 1 mM EDTA, 1% NP‐40 Substitute, and 0.05% SDS. Lysates were centrifuged at 15 300 × *g* for 10 min, and the supernatant was incubated with anti‐Myc or control IgG and Protein G Sepharose (GE Healthcare, cat. no. 17061801) for 4 h at 4°C. Immunocomplexes were pelleted, washed three times with lysis buffer, and resuspended in SDS sample buffer.

### 2.12 Separation of PSD Fraction and Lipidomics Analysis by LC–MS/MS

PSD fractions were isolated from WT and *reeler* mouse brains as previously described (Bermejo et al. 2014) with minor modifications. Brains were homogenized in ice‐cold 0.32 M sucrose containing 25 mM HEPES (pH 7.2). The homogenate was centrifuged at 900 × *g* for 10 min at 4°C. The supernatant was collected and centrifuged twice at 10 000 × *g* for 15 min. The resulting pellet was resuspended in sucrose buffer and loaded onto a discontinuous sucrose gradient, followed by ultracentrifugation at 150 000 × *g* for 2 h at 4°C. The synaptic plasma membrane fraction was collected from the lower interface, pelleted by ultracentrifugation at 200 000 × *g* for 30 min, resuspended in HEPES/EDTA buffer (50 mM HEPES, 2 mM EDTA, 0.5% Triton X‐100), and ultracentrifuged at 32 000 × *g* for 20 min. The final pellet was solubilized in buffer containing 50 mM HEPES, 2 mM EDTA, and 0.01% SDS. Lipids were extracted using the Bligh and Dyer method for subsequent LC–MS/MS analysis. LC–MS/MS analysis and annotation of MS/MS spectra were performed as previously described (Tsugawa et al. 2024). Briefly, the LC system consisted of a Waters Acquity ultraperformance LC (UPLC) system. Lipids were separated on an Acquity UPLC Peptide BEH C18 column (Waters). The column was maintained at 45°C and a flow rate of 0.3 mL min^−1^. The mobile phases consisted of (A) 1:1:3 (*v*/*v*/*v*) acetonitrile/methanol/water with 5 mM ammonium acetate and 10 nM EDTA and (B) 100% isopropanol with 5 mM ammonium acetate and 10 nM EDTA. Separation was conducted under the following gradient protocol: 0 min, 0% B; 1 min, 0% B; 5 min, 40% B; 7 min, 64% B; 12.5 min, 82.5% B; 19 min, 85% B; 20 min, 95% B; 20.1 min, 0% B; and 25 min, 0% B. MS detection of lipids was performed using a quadrupole/time‐of‐flight MS system (TripleTOF 6600, Sciex). All analyses were performed in high‐resolution mode in MS1 (~35 000 full width at half maximum) and high‐sensitivity mode in MS2 (~20 000 full width at half maximum). The parameters were as follows: MS1 and MS2 mass ranges, m/z 70–1250; MS1 accumulation time, 200 ms; MS2 accumulation time, 70 ms; collision energy, +40 eV/−42 eV; collision energy spread, 15 eV. The MS‐DIAL version 4.20 was used for analysis. The identification criteria were described previously (Tsugawa et al. 2024).

### 2.13 Statistical Analysis

All quantitative data are presented as mean ± SEM, with individual data points plotted. All statistical analyses were conducted using two‐tailed tests. Detailed information about statistical tests and methods is described in the respective figure legends. A priori sample size calculation was not performed; instead, the sample size and statistical analyses were determined based on previous similar studies with similar experimental designs (Kim et al. 2018; Kohno et al. 2024; Cabukusta et al. 2024). No blinding or test for outliers was performed. Given the limited number of replicates, we refrained from performing formal normality tests and interpreted parametric analyses with caution. All statistical analyses were performed using Microsoft Excel version 16.98 (Microsoft Corporation, RRID:SCR_016137) or Prism version 10.5.0 (GraphPad Software, RRID:SCR_002798).

### 2.14 Data Availability

The raw mass spectrometry data have been deposited in the MB‐POST database under accession number MPST000063 (https://repository.massbank.jp/entry/MPST000063). The datasets generated and/or analyzed during the current study are available from the corresponding author on reasonable request.

## 3 Results

### 3.1 A Specific SM Probe Visualizes SM in the Neuronal Plasma Membrane

**FIGURE 1:**
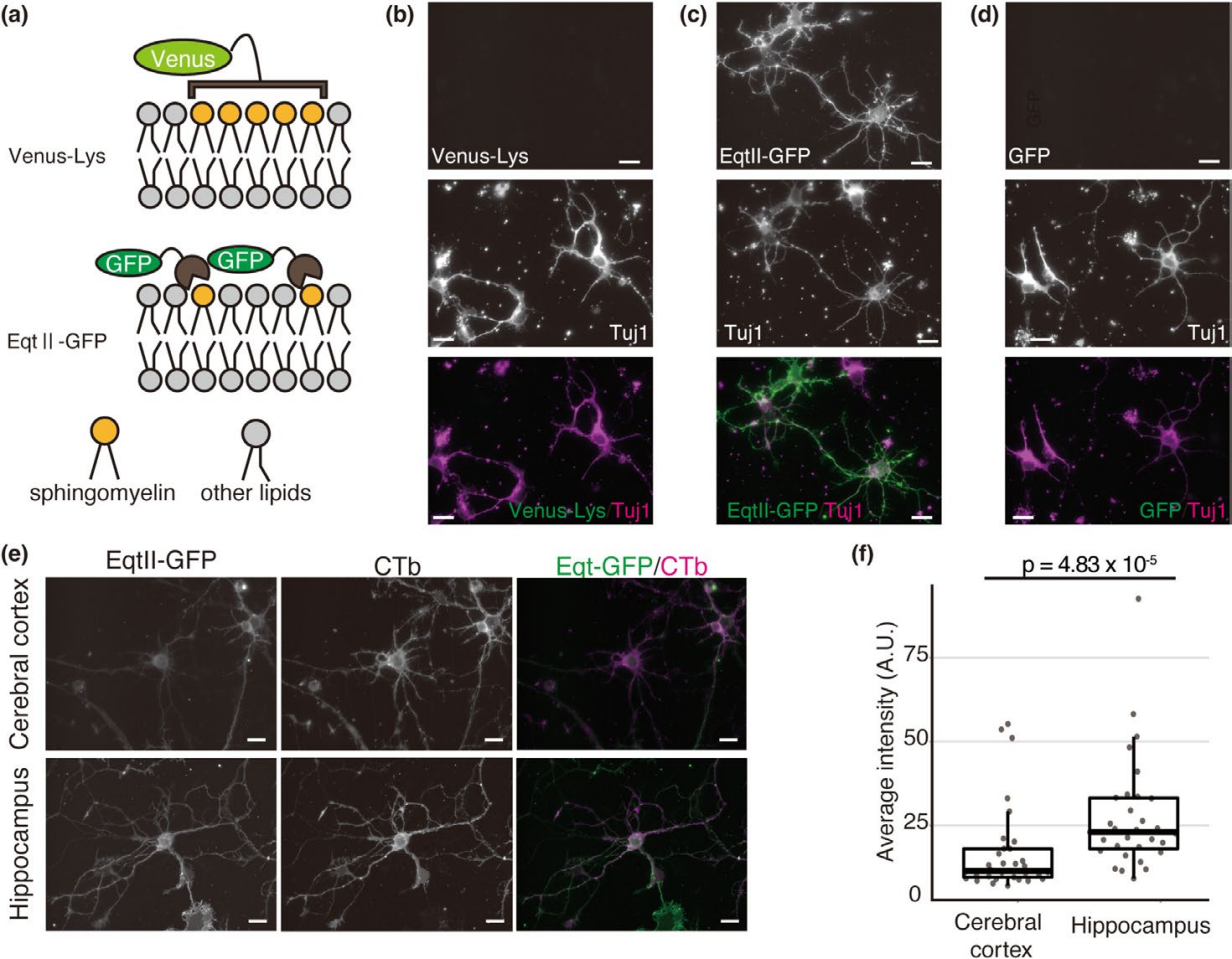
Visualization of SM on the plasma membrane of hippocampal and cerebral cortical neurons. (a) The schematic diagram illustrating the binding of an SM‐specific probe, Venus‐Lysenin (Venus‐Lys, left) and Equinatoxin II‐GFP (EqtII‐GFP, right). (b–d) Primary cultured hippocampal neurons were stained with Venus‐Lys (b), EqtII‐GFP (c), or GFP (d) (top panels). The cells were simultaneously stained with TuJ1 antibody for visualizing neuronal morphology (middle panels). Merged images are shown in the bottom. Scale bars = 20 μm. (e) Primary cultured cerebral cortical (upper panels) or hippocampal neurons (lower panels) were co‐stained with EqtII‐GFP and cholera toxin subunit B (CTb). Scale bars = 20 μm. (f) The boxplot showing the fluorescent intensity of EqtII‐GFP. Mann–Whitney *U*‐test (U = 209, *p* = 4.83 × 10^−5^) *n*_cerebral cortex_ = 33, *n*_hippocampus_ = 30 individual cells. Box plots indicate the median (center line), interquartile range (IQR, box), and 1.5 × IQR whiskers. Individual data points are overlaid.

There is currently limited information regarding the distribution and abundance of SM on the neuronal plasma membrane. Using Venus‐Lys, a probe that specifically binds to clustered SM (Makino et al. 2015; Hullin‐Matsuda et al. 2018) (Figure 1a), a previous study demonstrated that SM clusters are rarely observed in cultured hippocampal neurons (Kidani et al. 2012). Given that neuronal plasma membranes are known to be enriched in SM (Gaudioso et al. 2023; Westra et al. 2021), it was suggested that clusters composed solely of SM are scarce in cultured hippocampal neurons.

In the present study, we employed Equinatoxin II‐GFP (EqtII‐GFP), a probe that binds to individual SM molecules (Makino et al. 2015; Hullin‐Matsuda et al. 2018) (Figure 1a), to investigate the distribution of SM on the neuronal plasma membrane. Primary cultured hippocampal neurons were fixed with PFA and incubated with either Venus‐Lys or EqtII‐GFP without membrane permeabilization. Subsequently, neurons were permeabilized and immunostained with the Tuj1 antibody. As shown in Figure 1b–d, EqtII‐GFP successfully labeled the neuronal plasma membrane, whereas Venus‐Lys did not. These results confirm that SM is predominantly unclustered on the neuronal plasma membrane and can therefore be detected using EqtII‐GFP.

Previous reports have shown that the two isoforms of sphingomyelin synthase (SMS1 and SMS2) are differentially expressed among neuronal cell types in the central nervous system (Huitema et al. 2004; Kilkus et al. 2008; Kidani et al. 2012). To compare SM levels on the neuronal plasma membrane between different brain regions, we analyzed primary cultured neurons derived from the cerebral cortex and hippocampus of WT mice using EqtII‐GFP. As illustrated in Figure 1e,f, hippocampal neurons exhibited stronger EqtII‐GFP labeling than cortical neurons, suggesting a higher SM content on the plasma membrane of hippocampal neurons. Based on these findings, primary cultured hippocampal neurons were used in subsequent experiments.

### 3.2 Reelin Stimulation Increases SM Content in the Neuronal Plasma Membrane

**FIGURE 2:**
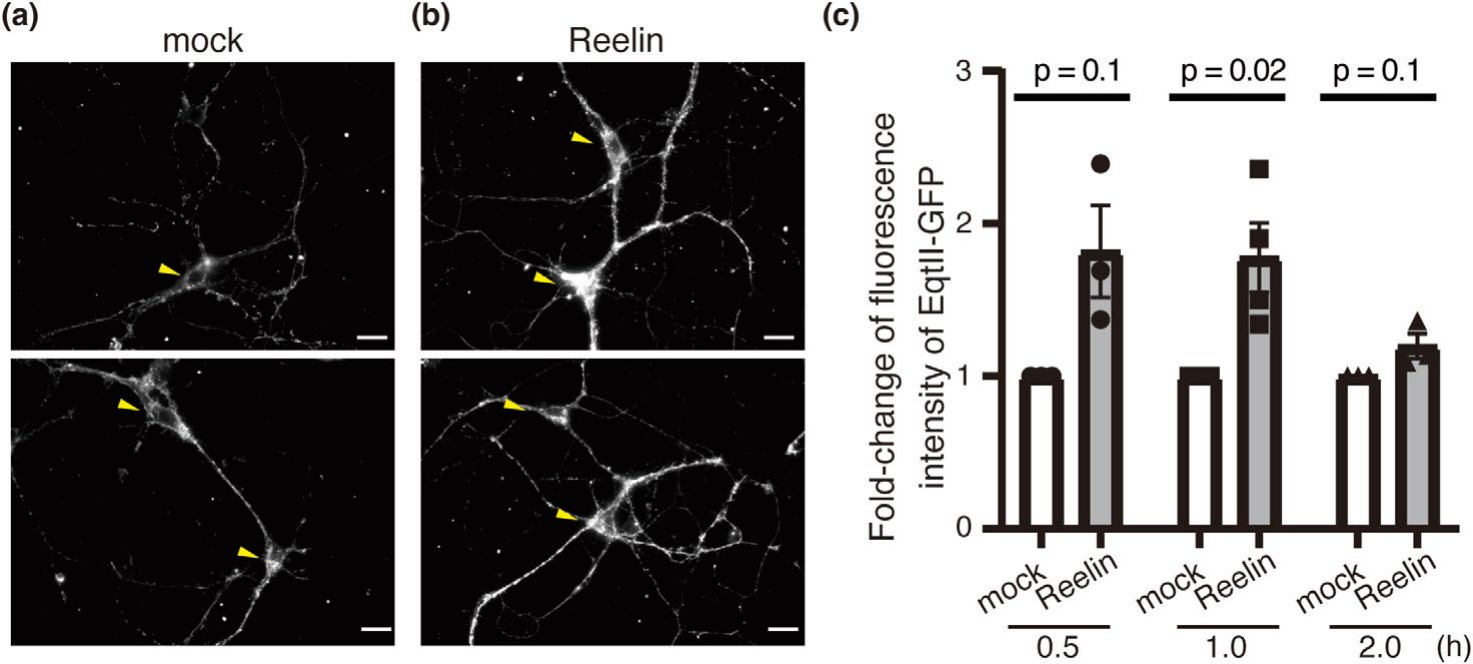
Reelin increases the SM content in the neuronal plasma membrane. (a, b) Images of primary cultured hippocampal neurons stained with EqtII‐GFP after incubation with control (mock, a) or Reelin‐containing (b) media. Yellow arrowheads indicate neuronal cell bodies. Scale bars = 20 μm. (c) Quantification of the fluorescent intensity of EqtII‐GFP. Each dot represents an independent set of experiment. Data are presented as means ± SEM. One‐sample *t*‐test (*n*_0.5 h_ = 3, *n*_1 h_ = 4, *n*_2 h_ = 3, independent cell culture preparations, 0.5 h, df = 2, *t* = −2.71, *p* = 0.113; 1 h, df = 3, *t* = −4.49, *p* = 0.0206; 2 h, df = 2, *t* = −2.37, *p* = 0.141) was performed.

We investigated the effect of Reelin on the SM content of the neuronal plasma membrane. To eliminate the influence of endogenous Reelin, primary hippocampal neurons were prepared from *reeler* mice and cultured for 7 days in vitro. The neurons were then stimulated with recombinant Reelin or control medium for 30 min, 1 h, or 2 h. Following stimulation, the neurons were fixed and stained with EqtII‐GFP to assess SM content. A significant increase in EqtII‐GFP binding was observed after 1 h of Reelin stimulation (Figure 2a,b). A trend toward increased EqtII‐GFP binding was also noted at 30 min and 2 h, although these changes did not reach statistical significance (Figure 2b). These findings suggest that Reelin transiently enhances SM content on the plasma membrane of hippocampal neurons.

### 3.3 Reelin Enhances SM Synthesis in Primary Cultured Hippocampal Neurons

**FIGURE 3:**
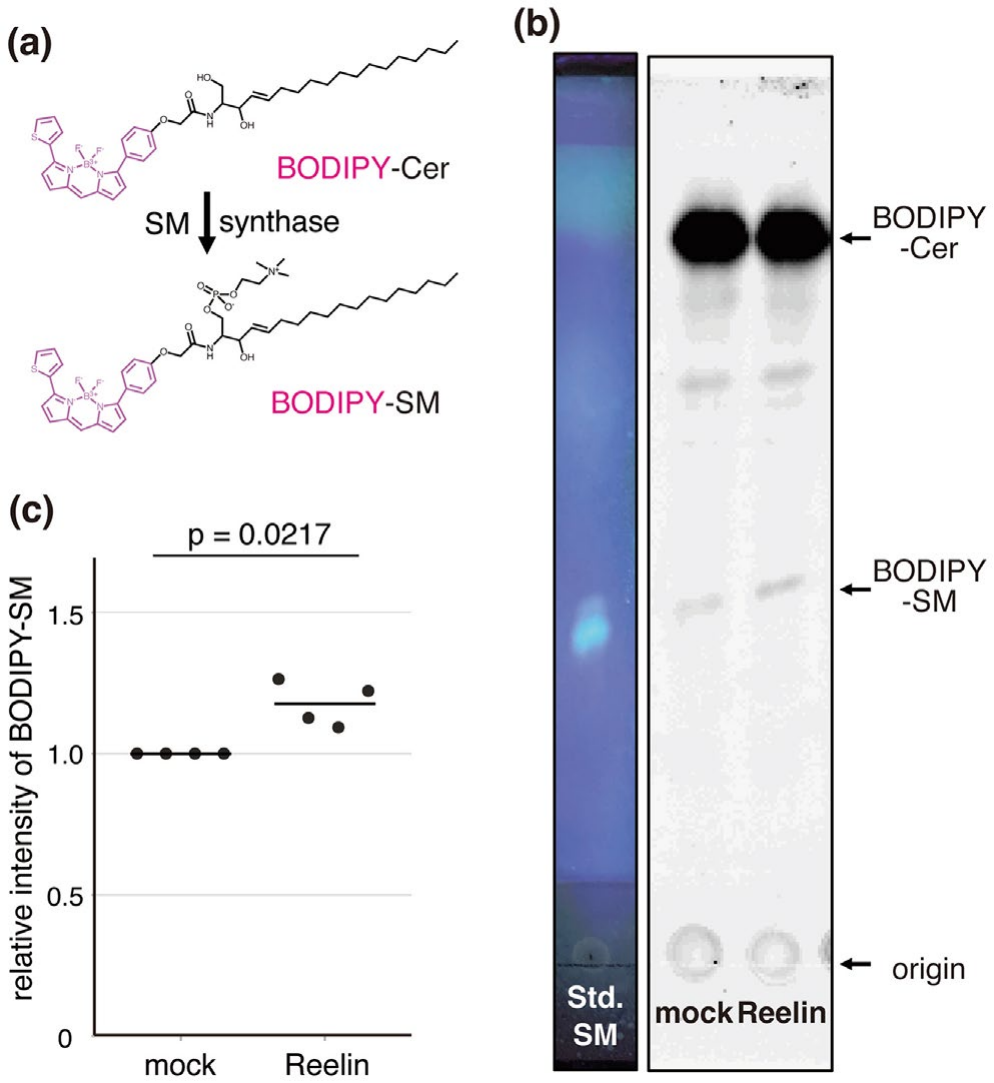
Reelin upregulates the SM synthesis in the primary cultured hippocampal neurons. (a) Reaction mediated by SM synthase. Magenta indicates BODIPY. (b) Thin layer chromatography results. Standard SM (Std. SM) was visualized using Primulin solution (left panel). Samples were developed on the same plate as the standard SM and detected by the fluorescence of BODIPY (right panel). (c) Quantification of BODIPY‐SM production. Each dot represents independent experiments (*n* = 4 independent cell culture preparations). One sample *t*‐test (df = 3, *t* = −4.40, *p* = 0.0217) was performed.

Previous studies in non‐neuronal cells have demonstrated that increased SM levels at the plasma membrane are attributed to enhanced SM synthesis capacity (Kim et al. 2018; Kidani et al. 2012). To determine whether Reelin similarly promotes SM synthesis in neurons, we employed a fluorescent metabolic labeling approach using BODIPY‐conjugated ceramide (BODIPY‐Cer), which is converted into BODIPY‐labeled SM (BODIPY‐SM) via the activity of SM synthases (Figure 3a). Primary hippocampal neurons were incubated for 1 h with either Reelin‐containing medium or control medium in the presence of BODIPY‐Cer. Following incubation, total lipids were extracted from the neurons, separated by TLC, and the amount of BODIPY‐SM was quantitated. The analysis revealed a marked increase in BODIPY‐SM levels in Reelin‐treated neurons compared to control neurons (Figure 3b,c), indicating enhanced SM synthesis upon Reelin stimulation. These findings suggest that Reelin promotes de novo SM synthesis in hippocampal neurons, potentially contributing to the observed increase in SM content at the plasma membrane.

### 3.4 Reelin Does Not Induce Phosphorylation of SM Synthase 2

**FIGURE 4:**
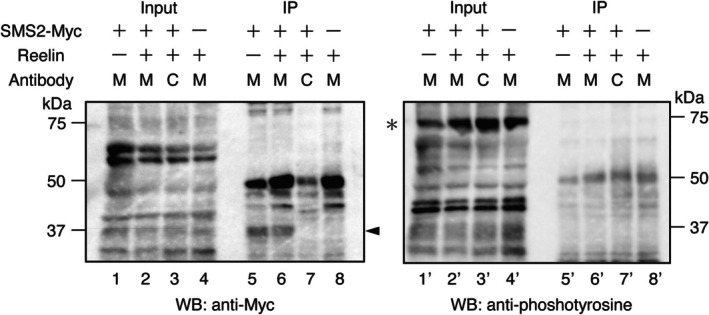
Reelin does not induce the tyrosyl phosphorylation of SMS2 in cortical neurons. Western blot analysis of tyrosine phosphorylation of SM synthase 2 (SMS2)‐Myc. Neurons transfected with expression vectors of SMS2‐Myc (lanes 1–3, 5–7, 1′‐3′, and 5′‐7′) or control GFP (lanes 4, 8, 4′, and 8′) were incubated with Reelin‐containing medium (lanes 2–4, 6–8, 2′‐4′, and 6′‐8′) or control medium (lanes 1, 5, 1′, and 5′) for 30 min. Neurons were then lysed and immunoprecipitations using anti‐Myc (lanes 1, 2, 4–6, 8, 1′, 2′, 4′‐6′, and 8′, indicated as “M”) or control IgG (lanes 3, 7, 3′, and 7′, indicated as “C”). The precipitated proteins were resolved with SDS‐PAGE and transferred to PVDF membrane, which was analyzed with an anti‐Myc antibody (left panel) and then with an anti‐phosphotyrosine antibody (right panel). Molecular mass markers (kDa) are shown on the left of each panel. Arrowhead and asterisk indicate SMS2‐Myc and Dab1, respectively.

In non‐neuronal cells, the Src family tyrosine kinase Fyn promotes the phosphorylation of SM synthase 2 (SMS2), which leads to the stabilization of SMS2 protein and the increase of SM amount (Baba et al. 2009; Kim et al. 2018). Given that Fyn is also activated by Reelin in neurons, we hypothesized that Reelin stimulation may induce SMS2 phosphorylation. To test this, we expressed Myc‐tagged SMS2 (SMS2‐Myc) in primary cortical neurons derived from *reeler* mice and stimulated the cells with either Reelin‐containing or control medium. Because the amount of plasma membrane SM tended to increase at 30 min, increased significantly at 1 h, and showed no change at 2 h after Reelin addition (Figure 2c), it was presumed that the SMS2 stabilization must be initiated within 30 min of Reelin incubation and taper off by 2 h. Therefore, we selected a 30‐min duration for Reelin stimulation. SMS2‐Myc was immunoprecipitated and subjected to western blot analysis. As a positive control for Reelin signaling activation, the phosphorylation of Dab1—a well‐established downstream effector of Reelin—was confirmed in whole cell lysates (Figure 4; asterisk in lanes 2′, 3′, and 4′, right panel). However, no phosphorylation of immunoprecipitated SMS2‐Myc was detected in Reelin‐treated neurons (Figure 4, lane 6′, right panel). These results indicate that Reelin‐induced enhancement of SM synthesis occurs independently of SMS2 phosphorylation. Reelin may regulate SM synthesis through a mechanism distinct from Fyn‐mediated SMS2 stabilization.

### 3.5 Reelin Enhances the Surface Expression of GPI‐Anchored Proteins on the Plasma Membrane in Hippocampal Neurons

**FIGURE 5:**
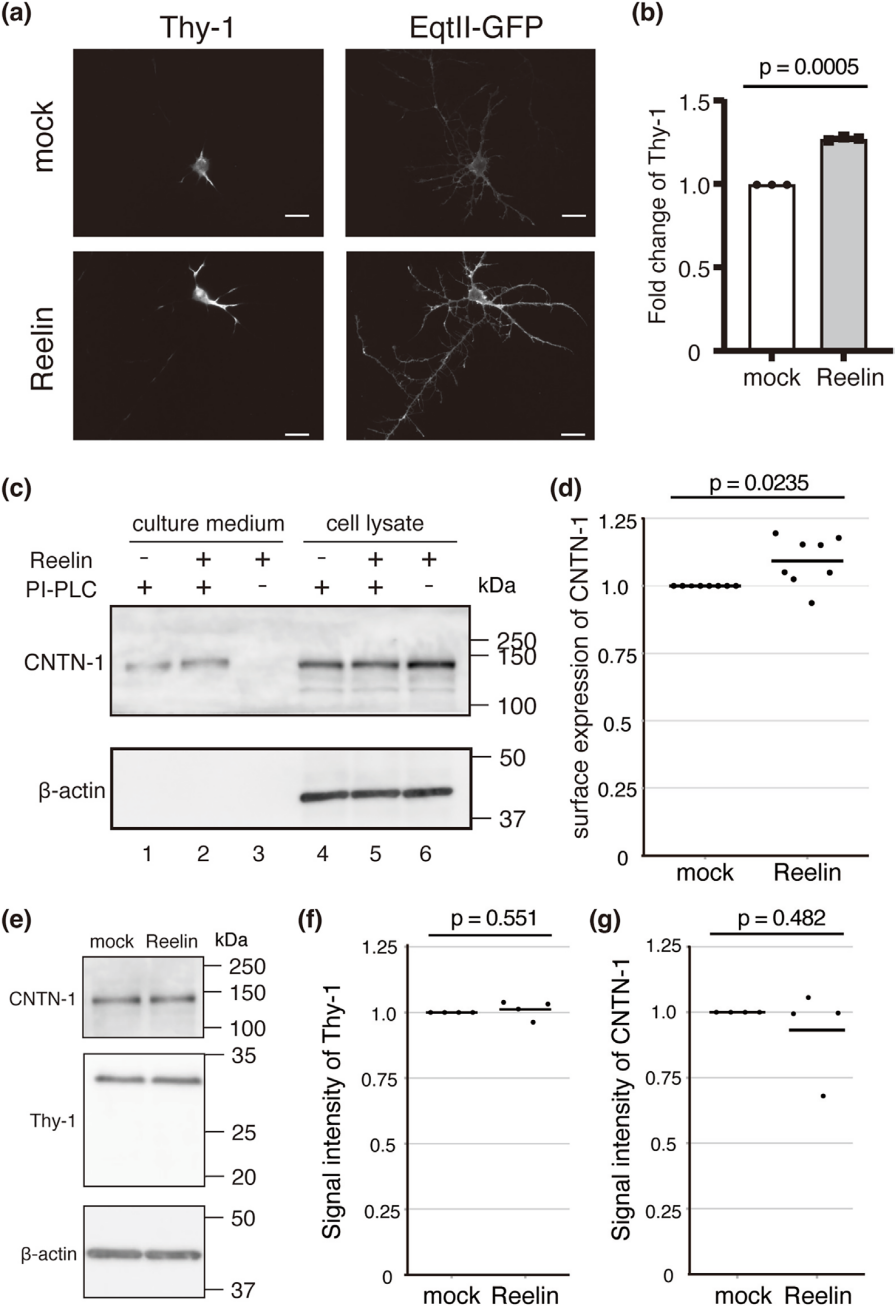
Reelin increases the surface expression of GPI‐anchored proteins on the plasma membrane in hippocampal neurons. (a) Hippocampal neurons were stained with an anti‐Thy‐1 antibody and EqtII‐GFP after treatment with mock or Reelin‐containing medium. Scale bars = 20 μm. (b) Quantification of the fluorescent intensity of Thy‐1. Each dot represents an independent experiment (*n* = 3 independent cell culture preparation). One‐sample *t*‐test (df = 2, *t* = −46.5, *p* = 4.61 × 10^−4^). (c) Western blot analysis of the culture supernatant (lanes 1–3) or the cell lysate (lanes 4–6) after Phosphatidylinositol‐specific phospholipase C (PI‐PLC) treatment of hippocampal neurons incubated with mock (lanes 1 and 4) or Reelin‐containing (lanes 2, 3, 5 and 6) medium. The primary antibodies used are shown on the left of the panels. Positions of molecular mass markers (kDa) are shown on the right of the panels. (d) Quantification of the surface expression ratio of contactin‐1 (CNTN‐1). Each dot represents an independent experiment (*n* = 8 independent cell culture preparations). One‐sample *t*‐test (df = 7, *t* = −2.88, *p* = 0.0235) was performed. (e) Western blot analysis of Reelin influence on the total amount of Thy‐1 or CNTN‐1 in the neurons. Molecular mass markers (kDa) are shown on the right of each panel. (f) Quantification of the signal of Thy‐1 normalized with β‐actin signal. Each dot represents an independent experiment (*n* = 4 independent cell culture preparations). One‐sample *t*‐test was performed. (g) Quantification of the signal of CNTN‐1 normalized with β‐actin signal. Each dot represents an independent experiment (*n* = 4 independent cell culture preparations). One‐sample *t*‐test (Thy‐1, df = 3, *t* = −0.670, *p* = 0.551; CNTN‐1, df = 3, *t* = 0.801, *p* = 0.482) was performed in (f) and (g).

We next sought to investigate the impact of Reelin‐induced increases in SM levels on neuronal membranes. SM contributes to the formation of microdomains often called lipid rafts on the plasma membrane, which regulate the localization and function of various proteins (Lingwood and Simons 2010). Notably, glycosylphosphatidylinositol (GPI)‐anchored proteins are known to concentrate within these lipid rafts. Therefore, an increase in SM may expand the area of lipid rafts, potentially enhancing the surface expression of GPI‐anchored proteins. To test this hypothesis, we examined the surface levels of two representative GPI‐anchored proteins, Thy‐1 and Contactin‐1 (CNTN‐1), in hippocampal neurons. Immunostaining of neurons derived from *reeler* mice using anti‐Thy‐1 antibody under non‐permeabilized conditions revealed that Reelin stimulation significantly increased the surface expression of Thy‐1 (Figure 5a,b). In the case of CNTN‐1, its high basal surface abundance hindered accurate quantification by immunostaining (data not shown). Thus, we employed phosphatidylinositol‐specific phospholipase C (PI‐PLC) to assess surface expression levels. Following Reelin stimulation, neurons were treated with PI‐PLC, and both the culture medium and cell lysates were analyzed by western blotting. Reelin treatment led to a marked increase in the proportion of CNTN‐1 released into the medium (Figure 5c,d), indicating enhanced surface expression. Importantly, Reelin stimulation did not alter the total cellular levels of either Thy‐1 or CNTN‐1 (Figure 5e–g). These findings suggest that Reelin promotes the surface expression of GPI‐anchored proteins on the plasma membrane, potentially via an SM‐mediated expansion of lipid raft domains.

### 3.6 The Composition of Lipid Species Involved in SM Metabolism Was Aberrant in the PSD Fraction of *Reeler* Mice

**FIGURE 6:**
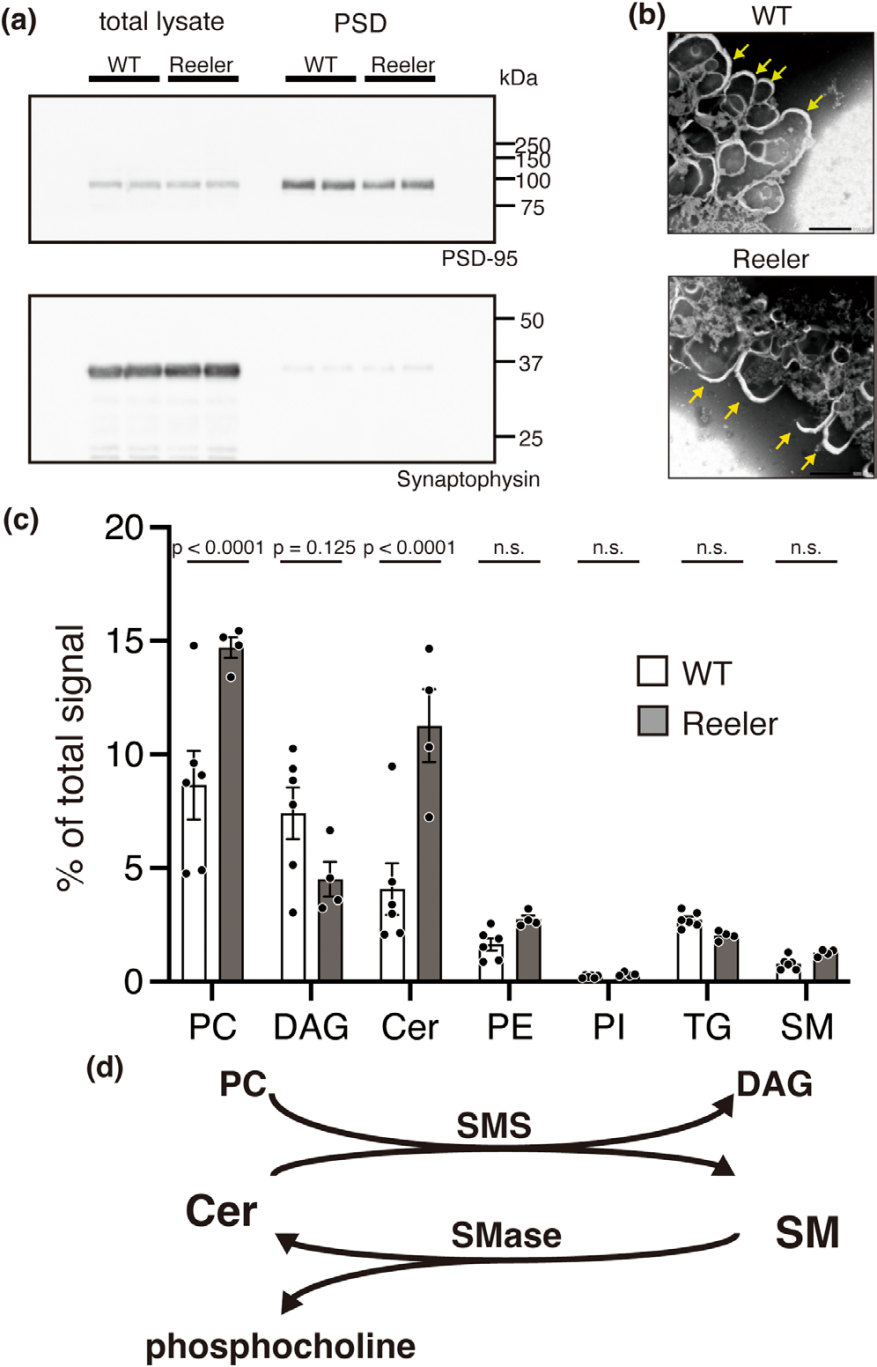
The composition of lipid species involved in SM metabolism was aberrant in the PSD fraction of *reeler* mice. (a) Validation of the fractions was performed by western blotting. (b) Transmission electron microscopy images of isolated postsynaptic density region (PSD). Yellow arrows indicate plasma membrane components in PSD. Scale bars = 500 nm. (c) The ratios of each lipid species analyzed by LC–MS/MS. Cer, ceramide; DAG, diacylglycerol; PC, phosphatidylcholine; PE, phosphatidylethanolamine; PI, phosphatidylinositol; TG, triglyceride. Each dot represents one sample (*n*_WT_ = 6, *n*_reeler_ = 4, independently prepared samples from independent mice for each genotype). Two‐way ANOVA was performed (F_6,56_ = 9.496, *p* = 3.46 × 10^−7^), followed by Bonferroni's multiple comparisons test (PC, *t* = 5.10, *p* = 2.96 × 10^−5^; DAG, *t* = 2.44, *p* = 0.125; Cer, *t* = 6.052, *p* = 8.76 × 10^−7^; PE; *t* = 0.933, *p* = 2.48; PI, *t* = 0.0890, *p* = 6.51; TG, *t* = 0.592, *p* = 3.89; SM, *t* = 0.394, *p* = 4.87. The degree of freedom of each lipid is 56). (d) Reaction equation for SM metabolism.

We postulated that examining the postsynaptic density (PSD) would be a good approach to test whether Reelin also increases the SM level in neuronal cell membranes in vivo, because PSD is one of the targets of Reelin in vivo (Jossin 2020; Ventruti et al. 2011; Wasser and Herz 2017), as it can be isolated by biochemical methods and only includes neuronal cell membrane. We performed lipidomic analysis on the PSD fraction from WT and *reeler* mice. Validation was done via western blotting with anti‐PSD‐95 and anti‐synaptophysin antibodies (Figure 6a) and transmission electron microscopy (Figure 6b). Lipids were extracted and analyzed using LC–MS/MS. We found that the levels of phosphatidylcholine (PC) and ceramide (Cer), the substrates of SM synthesis (Figure 6d), were higher in the PSD of *reeler* mice. Concomitantly, although the difference did not reach statistical significance, the level of DAG, a molecule that occurs simultaneously with SM during SM synthesis (Figure 6d), tended to be lower in the PSD of *reeler* mice (Figure 6c). SM was not reliably detected, and its level did not significantly differ between WT and *reeler* mice (Figure 6c). Nonetheless, these results may indicate that the absence of Reelin resulted in the decrease of SM synthesis in the PSD.

## 4 Discussion

In this study, we demonstrate that Reelin signaling enhances SM content in the plasma membrane of hippocampal neurons by promoting de novo SM synthesis. Using a monomer‐specific SM probe EqtII‐GFP, we visualized the distribution of SM in neurons and found that Reelin stimulation significantly increased SM levels. Furthermore, comparative lipidomic analysis and metabolic labeling with BODIPY‐Cer supported that Reelin enhances SM biosynthesis in neurons. Together, these results unveil a novel mechanism by which Reelin regulates neuronal membrane lipid composition, adding an important layer to the multifaceted roles of Reelin in brain development and synaptic function.

Previous reports have largely focused on the canonical signaling cascade initiated by Reelin binding to ApoER2 and VLDLR, leading to Dab1 phosphorylation and downstream activation of pathways such as PI3K/Akt, Src family kinases, and LIMK‐cofilin signaling (Bock and Herz 2003; Herz and Chen 2006). While these pathways have been implicated in cytoskeletal remodeling and synaptic plasticity, relatively little attention has been paid to how Reelin might modulate the lipid environment of the neuronal plasma membrane, despite the crucial role of membrane lipids in organizing signaling platforms (Lingwood and Simons 2010). Our findings that Reelin enhances SM biosynthesis provide compelling evidence that lipid metabolism is a previously underappreciated downstream effector of Reelin signaling. It is important to point out that SM content in the primary cortical neurons is generally lower than that in hippocampal neurons (Figure 1e,f). In fact, Reelin treatment did not enhance SM synthesis in cortical neurons to the same extent as in hippocampal neurons (Figure S1a,b). While elucidating the basis for this discrepancy will require further investigation, several intriguing possibilities can be considered. First, cholesterol depletion—which disrupts SM/cholesterol‐rich microdomains—has been reported to promote neurite outgrowth in hippocampal neurons but not in cortical neurons (Ko et al. 2005), and cholesterol‐dependent axon outgrowth in hippocampal neurons has been shown to rely on Fyn activity (Ko et al. 2005). These findings suggest that the signaling pathways downstream of Fyn, and of its upstream regulator Reelin, may differ substantially between hippocampal and cortical neurons. Second, primary cultured neurons from the hippocampus and cortex have been reported to differ in both the time course of neurite outgrowth and the peak expression levels of synaptic markers (Kim and Lee 2012). Therefore, it is conceivable that differences in SM synthesis could be observed if neurons at different culture stages are compared, as distinct sets of proteins are expressed at different developmental stages.

One important implication of our study is that Reelin‐mediated SM upregulation may influence the localization and function of GPI‐anchored proteins. SM is believed to localize predominantly in the outer leaflet of the plasma membrane (but also see Mori et al. 2024) and to contribute to the formation of specialized domains such as lipid rafts (Lingwood and Simons 2010). GPI‐anchored proteins are known to preferentially localize to such domains (Paulick and Bertozzi 2008). We observed increased surface expression of GPI‐anchored proteins Thy‐1 and CNTN‐1 following Reelin stimulation, suggesting that the Reelin‐induced elevation of SM levels facilitates the stabilization or trafficking of these proteins on the neuronal surface. Given that both Thy‐1 and CNTN‐1 are implicated in neurite outgrowth, synaptic adhesion, and plasticity (Rege and Hagood 2006; Shimoda and Watanabe 2009), their enhanced surface localization may contribute to Reelin's roles in dendritic maturation and synaptic modulation.

Interestingly, although Reelin stimulation increased SM synthesis, we did not detect phosphorylation of SMS2, suggesting that Reelin regulates SM biosynthesis through a mechanism distinct from direct phosphorylation of SMS2. In non‐neuronal cells, Fyn‐mediated phosphorylation of SMS2 has been shown to increase its stability (Kim et al. 2018), and, to the best of our knowledge, this is the only example of SMS regulation. However, in our system, alternative regulatory mechanisms—such as transcriptional upregulation of SM synthases, post‐transcriptional modifications such as GRASP proteins (Calvo‐Jiménez et al. 2025), or modulation of ceramide availability—may be responsible. To the best of our knowledge, no antibody capable of reliably detecting endogenous SMS2 protein in tissues is currently available. Consequently, it is technically challenging to analyze changes in SMS2 expression levels or post‐translational modifications. Future studies investigating changes in SMS1/2 expression levels, subcellular localization, and enzymatic activity after Reelin stimulation will be crucial in delineating the exact molecular link between Reelin signaling and SM biosynthesis.

Our lipidomic analysis of PSD fractions further supports the notion that Reelin deficiency leads to impaired SM metabolism in vivo. The PSD is a highly specialized membrane domain that relies on precise lipid and protein composition for its structural and functional integrity (Emes and Grant 2012). Alterations in SM‐related lipid species in *reeler* mice could disrupt raft‐associated signaling complexes and impair synaptic function. These results may partially explain why Reelin signaling deficiency is associated with synaptic deficits observed in neurodevelopmental and neurodegenerative disorders such as schizophrenia and Alzheimer's disease (Folsom and Fatemi 2013; Krstic et al. 2013; Cuchillo‐Ibañez et al. 2016; Faini et al. 2021; Reive et al. 2024). However, in our lipidomic analysis, no significant changes were observed in the amount of SM itself. This may be partly because only two SM species (SM 36:1;2O and SM 38:1;2O) were detected. It is possible that the detergent used during the isolation of the PSD fraction increased the solubility of SM, resulting in incomplete recovery during lipid extraction. Alternatively, SM may be supplied to neurons from astrocytes and other glial cells (Barber and Raben 2019; Fitz et al. 2021), thereby maintaining a relatively constant SM level. To fully understand how Reelin affects the composition of SM in the PSD fraction, a more detailed lipid analysis is necessary. This should include specific methods for extracting and analyzing SM, as well as improved techniques for compartmentalizing the PSD fraction.

The discovery that Reelin influences membrane lipid composition opens new perspectives on how extracellular cues can orchestrate intracellular events through membrane remodeling. Membrane lipids not only serve structural functions but actively participate in receptor clustering, endocytosis, and signal transduction (Lingwood and Simons 2010; Girych et al. 2023). By modulating SM levels, Reelin may fine‐tune membrane microdomain organization, thereby optimizing the localization and efficiency of signaling complexes essential for neuronal development and plasticity.

In conclusion, our study identifies SM biosynthesis as a novel downstream target of Reelin signaling in neurons. This finding expands the functional repertoire of Reelin beyond cytoskeletal and synaptic regulation to include lipid metabolism. Understanding the interplay between Reelin signaling and membrane lipid homeostasis will provide deeper insights into the molecular mechanisms underlying brain development and the pathogenesis of neuropsychiatric and neurodegenerative disorders. Future investigations should aim to clarify how disruptions in this lipid regulatory pathway contribute to disease phenotypes and whether targeting SM metabolism could offer new therapeutic strategies.

## Author Contributions

**Yuto Takekoshi:** conceptualization, investigation, writing – original draft, methodology, validation, writing – review and editing, formal analysis, data curation, funding acquisition. **Hugo Ando:** investigation, methodology, formal analysis, data curation, writing – review and editing. **Takao Kohno:** investigation, methodology, data curation, writing – review and editing. **Hiroshi Takase:** investigation, methodology. **Tomohiko Taguchi:** methodology, investigation, resources. **Makoto Arita:** methodology, investigation, validation, formal analysis, data curation. **Toshihide Kobayashi:** resources, methodology, investigation. **Mitsuharu Hattori:** conceptualization, investigation, funding acquisition, writing – original draft, writing – review and editing, validation, visualization, methodology, formal analysis, project administration, data curation, supervision.

## Conflicts of Interest

The authors declare no conflicts of interest.

## Peer Review

The peer review history for this article is available at https://www.webofscience.com/api/gateway/wos/peer‐review/10.1111/jnc.70225.

## Supporting information


**Data S1:** jnc70225‐sup‐0001‐DataS1.pdf.

## Data Availability

The data that support the findings of this study are available from the corresponding author upon reasonable request.
