# Supplementary material for: Reelin Increases the Sphingomyelin Content of the Plasma Membrane and Affects the Surface Expression of GPI‐Anchored Proteins in Hippocampal Neurons
Source: J Neurochem. 2025 Sep 3;169(9):e70225. doi: 10.1111/jnc.70225 (PMC12406644; doi:10.1111/jnc.70225)
Supplement: Supplementary file 1 — Data S1: jnc70225‐sup‐0001‐DataS1.pdf. [file JNC-169-0-s001.pdf]

Reelin increases the sphingomyelin content of the plasma membrane and affects the surface expression of GPI-anchored proteins in hippocampal neurons

Yuto Takekoshi, Hugo Ando, Takao Kohno, Hiroshi Takase, Tomohiko Taguchi, Makoto Arita, Toshihide Kobayashi, and Mitsuharu Hattori

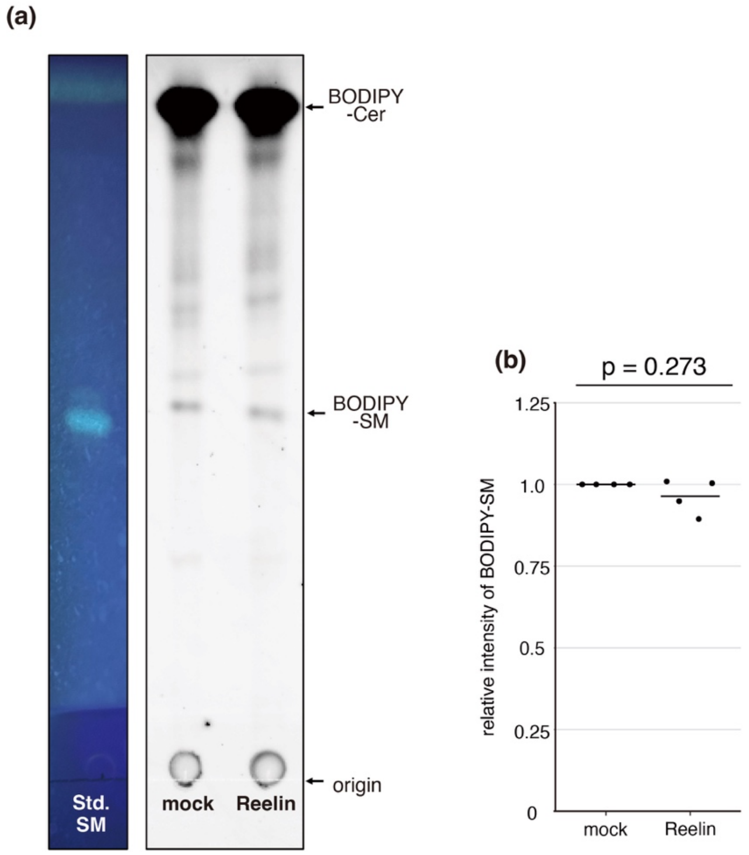

Supplementary FIGURE 1

Reelin does not increase SM synthesis ability in cortical neurons. (a) Thin layer chromatography results. Standard SM (Std. SM) was visualized using Primulin solution (left panel). Samples were developed on the same plate as the standard SM and detected by the fluorescence of BODIPY (right panel). (c) Quantification of BODIPY-SM production. Each dot represents independent data (n = 4 neurons from distinct mouse embryos). One sample t-test (df =3, t = 1.34, P = 0.273) was performed.

Reelin increases the sphingomyelin content of the plasma membrane and affects the surface expression of GPI-anchored proteins in hippocampal neurons

Yuto Takekoshi, Hugo Ando, Takao Kohno, Hiroshi Takase, Tomohiko Taguchi, Makoto Arita, Toshihide Kobayashi, and Mitsuharu Hattori

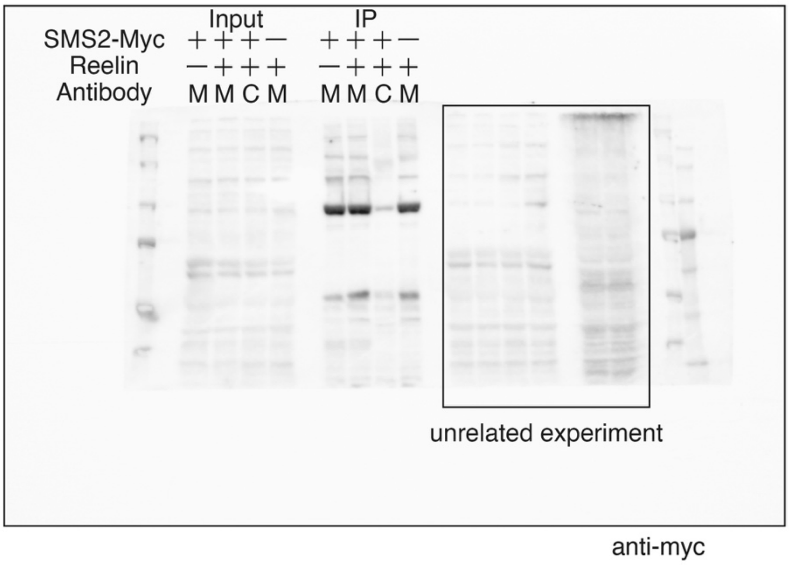

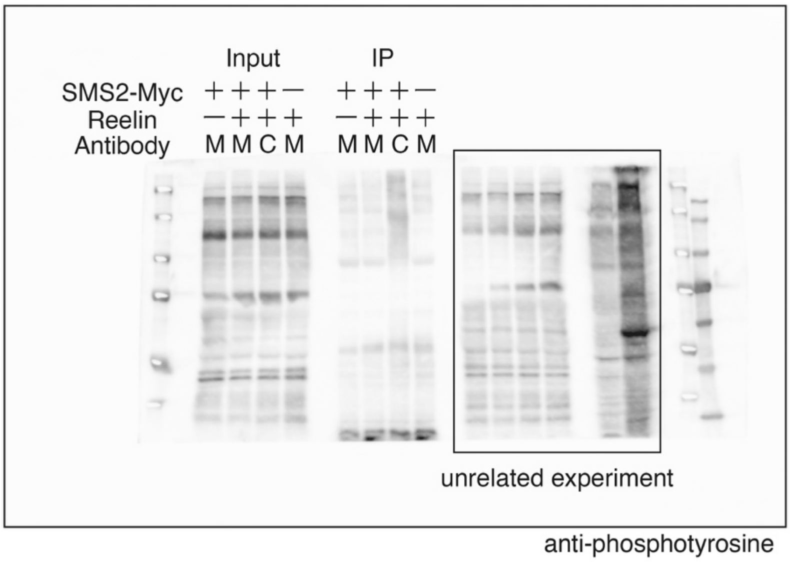

Supplementary FIGURE S2
Original uncropped western blotting data for FIGURE 4. Lanes unrelated to the current study are indicated as “unrelated experiment”.

Reelin increases the sphingomyelin content of the plasma membrane and affects the surface expression of GPI-anchored proteins in hippocampal neurons

Yuto Takekoshi, Hugo Ando, Takao Kohno, Hiroshi Takase, Tomohiko Taguchi, Makoto Arita, Toshihide Kobayashi, and Mitsuharu Hattori

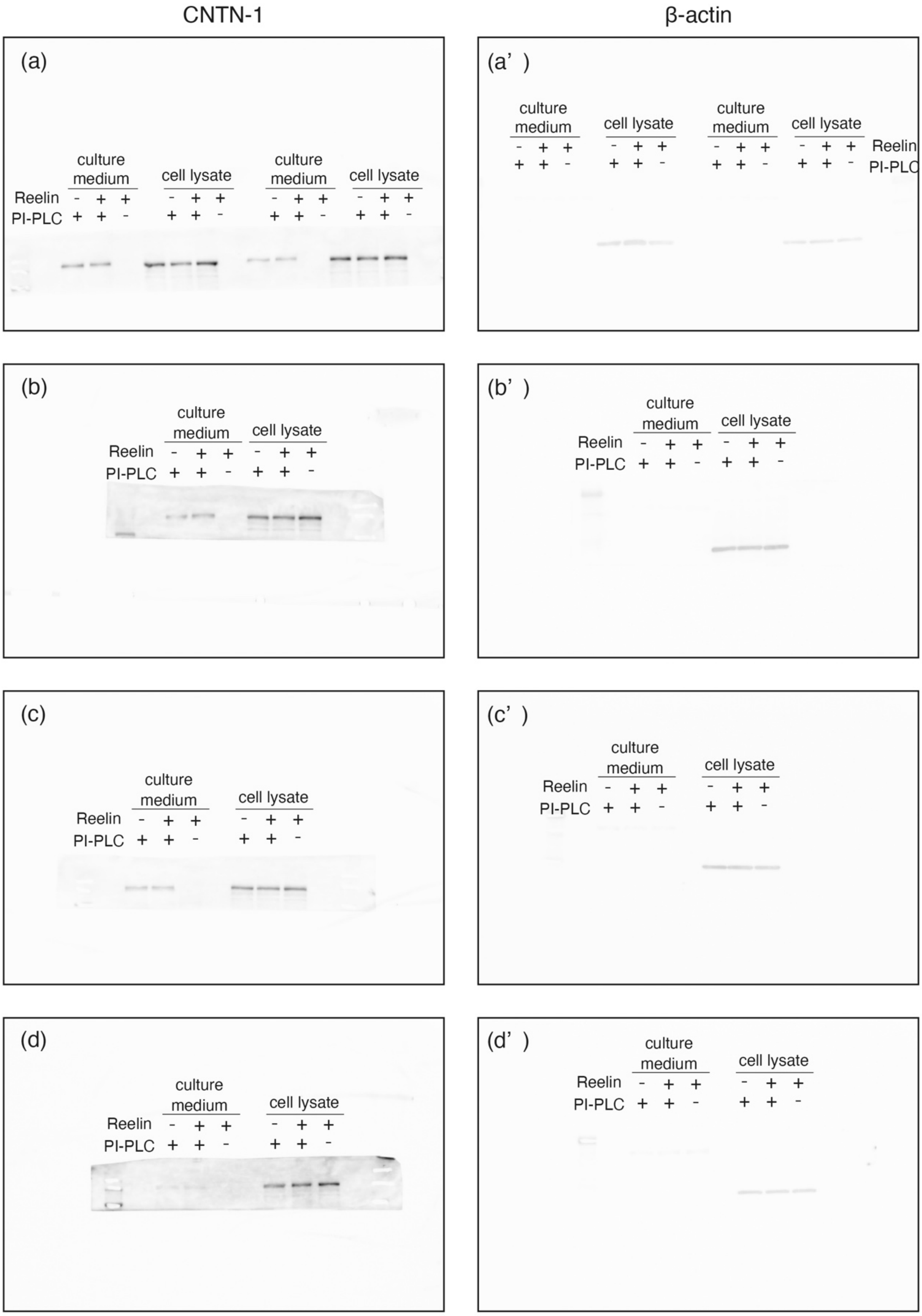

Reelin increases the sphingomyelin content of the plasma membrane and affects the surface expression of GPI-anchored proteins in hippocampal neurons

Yuto Takekoshi, Hugo Ando, Takao Kohno, Hiroshi Takase, Tomohiko Taguchi, Makoto Arita, Toshihide Kobayashi, and Mitsuharu Hattori

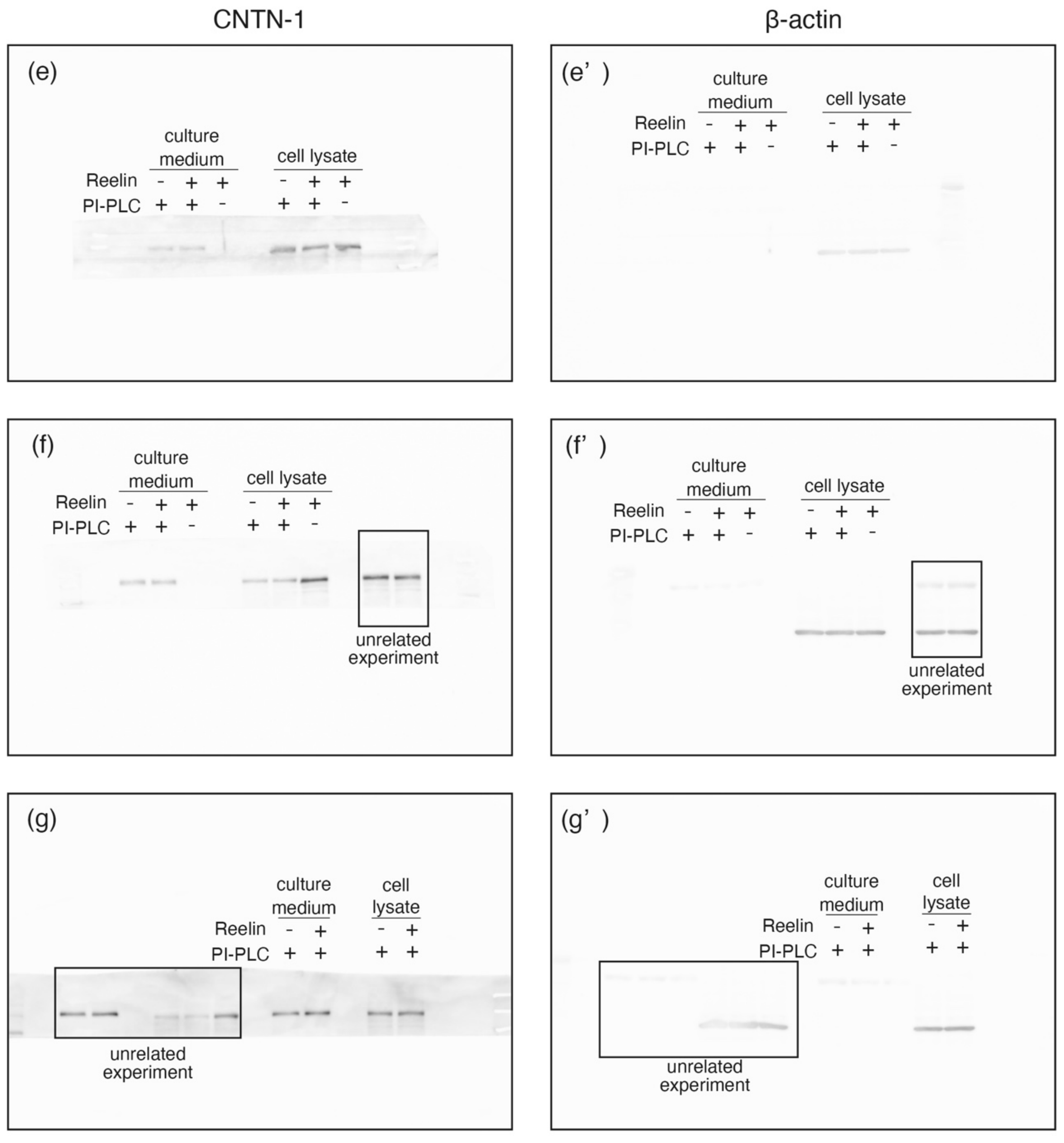

Supplementary FIGURE S3 Uncropped western blotting data for FIGURE 5c.

Original uncropped western blotting data for FIGURE 5c. a-g, CNTN-1; a'-g', β-actin. Lanes unrelated to the current study are indicated as “unrelated experiment”.

Reelin increases the sphingomyelin content of the plasma membrane and affects the surface expression of GPI-anchored proteins in hippocampal neurons

Yuto Takekoshi, Hugo Ando, Takao Kohno, Hiroshi Takase, Tomohiko Taguchi, Makoto Arita, Toshihide Kobayashi, and Mitsuharu Hattori

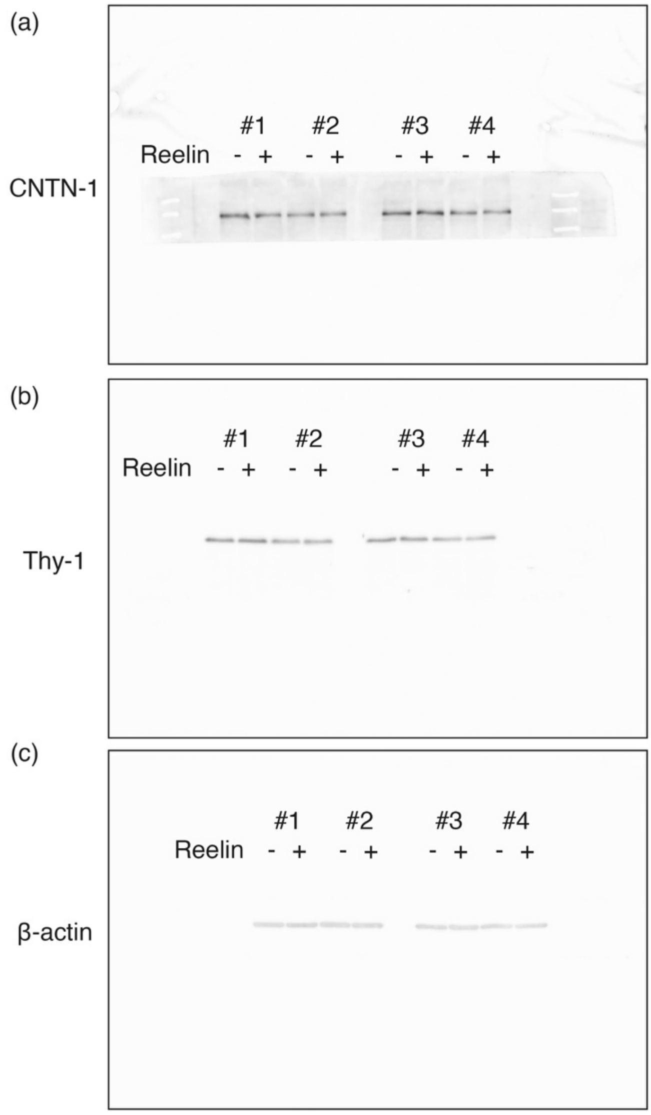

Supplementary FIGURE S4 Original uncropped western blotting data for FIGURE 5-e. Lanes with the same number are from the same sample, each from independent experiments.

Reelin increases the sphingomyelin content of the plasma membrane and affects the surface expression of GPI-anchored proteins in hippocampal neurons

Yuto Takekoshi, Hugo Ando, Takao Kohno, Hiroshi Takase, Tomohiko Taguchi, Makoto Arita, Toshihide Kobayashi, and Mitsuharu Hattori

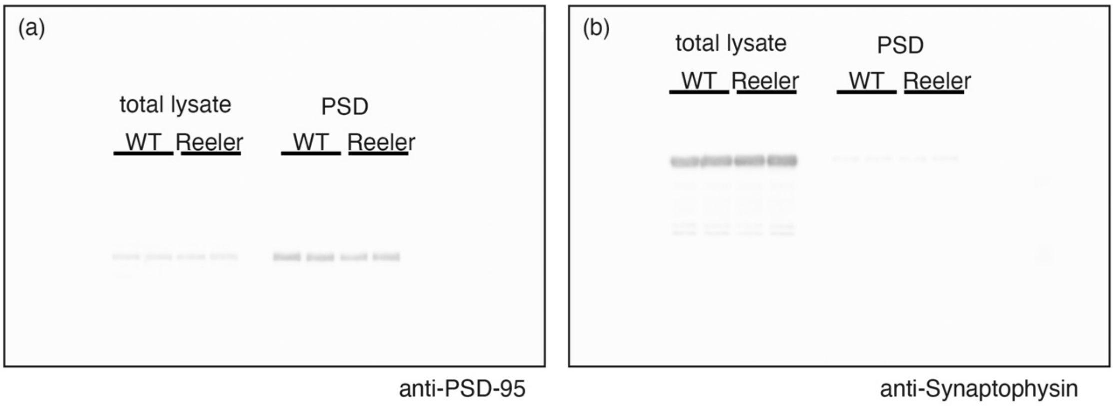

Supplementary FIGURE S5 Uncropped western blotting data for FIGURE 6.
